# The Efficacy and Safety of Cytarabine on Newly Diagnosed Primary Central Nervous System Lymphoma: A Systematic Review and Meta-Analysis

**DOI:** 10.3389/fonc.2020.01213

**Published:** 2020-07-31

**Authors:** Xiaohong Zheng, Shoubo Yang, Feng Chen, Si Wu, Wenbin Li

**Affiliations:** ^1^Department of Neuro-Oncology, Beijing Tiantan Hospital, Capital Medical University, Beijing, China; ^2^School of Management and Economics, Beijing Institute of Technology, Beijing, China

**Keywords:** primary central nervous system lymphoma, cytarabine, meta-analysis, chemotherapy, brain tumor

## Abstract

**Background:** The role of cytarabine on newly diagnosed primary central nervous system lymphoma (PCNSL) remains controversial. The present study mainly aimed to assess the efficacy and safety of cytarabine in the induction treatment of PCNSL.

**Methods:** We systematically searched PubMed, Embase, and the Cochrane library for randomized controlled trials comparing treatment of PCNSL patients with or without cytarabine. A meta-analysis was conducted to compare the odds ratios (ORs) with corresponding 95% confidence intervals (95% CI) for complete remission (CR) rate, overall response rate (ORR), grade 3–4 toxic effects, hazard ratios (HRs) with 95% CIs for progression-free survival (PFS), and overall survival (OS) using Stata 12.0.

**Results:** In total, three randomized clinical trials were analyzed in this study. The result of our statistical analysis demonstrated that the application of cytarabine was closely correlated with a higher CR (OR: 2.27, 95% CI: 1.29–3.99, *P* < 0.01) and ORR (OR: 2.11, 95% CI: 1.14–3.93, *P* = 0.02). No significant difference was found in OS (HR: 0.75, 95% CI: 0.50–1.13, *P* = 0.17), but PFS had been improved (HR: 0.66, 95% CI: 0.45–0.97, *P* = 0.04) when cytarabine was added to the treatment regimen. The grade 3–4 side effect rate of the cytarabine group was higher (overall OR: 2.95, 95% CI: 1.37–6.34, *P* < 0.01) than that of the cytarabine-free group.

**Conclusions:** This meta-analysis verifies that adding cytarabine to the therapeutic regimen is helpful for newly diagnosed PCNSL patients in terms of CR, ORR, and PFS. Moreover, it should be noted that the grade 3–4 toxic effects, especially hematological toxicity, are higher in the cytarabine group than in the cytarabine-free group. The results indicate that cytarabine plays an important role in the induction therapy of PCNSL. Large-sample and high-quality RCTs should be conducted to verify our results and confirm the effects of cytarabine on newly diagnosed PCNSL.

## Introduction

Primary central nervous system lymphoma (PCNSL) is a type of extranodal lymphoma exclusively occurring within the central nervous system, including the brain parenchyma, meninges, cranial nerves, eyes, and/or spinal cord. PCNSL accounts for 1–2% of all non-Hodgkin's lymphomas (NHLs) and for 2–7% of all primary central nervous system tumors (1). As an aggressive non-Hodgkin lymphoma, it usually demonstrates large B-cell histology (2). Since 2000, there has been an increase in the overall incidence of PCNSL, especially in the elderly (3).

The optimal treatment approach of PCNSL has yet to be established. Chemotherapy with high-dose methotrexate (HD-MTX) followed by whole-brain radiotherapy used to be the most common approach for patients with newly diagnosed PCNSL, resulting in a 5-year survival of 20–35% (4). A recent randomized controlled trial investigating the role of whole-brain radiotherapy (WBRT) as a consolidation therapy compared with non-consolidation therapy has suggested that WBRT does not prolong survival but enhances disease control (5). Several drugs have been recommended to be combined with HD-MTX to improve the outcome, such as cytarabine, a pyrimidine anti-metabolite acting on the proliferative S-phase of cells, because of the significant improvements in the treatment of systemic lymphoma. However, we found some conflicting reports about the role of cytarabine in the induction therapy of PCNSL. An international randomized trial (4) demonstrated that the combination of HD-MTX and cytarabine resulted in consistently better outcomes and acceptable toxicity compared with HD-MTX alone. However, some other studies (6, 7) showed that the non-cytarabine group appeared to be safer and more effective for PCNSL patients. Thus, the role of cytarabine in the induction therapy of PCNSL still needs to be elucidated.

In the current study, we conducted a systematic and quantitative meta-analysis using the available data regarding complete remission (CR) rate, overall response rate (ORR), grade 3–4 toxic effect rate, progression-free survival (PFS), and overall survival (OS) to assess the efficacy of treatment with or without cytarabine for PCNSL.

## Methods

### Search Strategy

This systematic review and meta-analysis is registered with the International Prospective Register of Systematic Reviews (number CRD42019131539).

We carefully searched academic databases (PubMed, EMBASE, and Cochrane Library) to identify relevant studies from the date the database was established till December 31, 2019. The search typically included two key terms “Primary central nervous system lymphoma” and “Cytarabine.” The complete search used for all database was (Arabinosylcytosine [Title/Abstract]) OR Cytosine Arabinoside [Title/Abstract]) OR Arabinoside, cytosine [Title/Abstract]) OR Arabinofuranosylcytosine [Title/Abstract]) OR Aracytidine [Title/Abstract]) OR beta-Ara C [Title/Abstract]) OR beta-Ara C [Title/Abstract]) OR Cytarabine Hydrochloride [Title/Abstract]) OR Cytosar [Title/Abstract]) OR Cytosar-U [Title/Abstract]) OR Cytosar U [Title/Abstract]) OR Ara-C [Title/Abstract]) OR Ara C [Title/Abstract]) OR Aracytine [Title/Abstract]) OR Cytonal [Title/Abstract])) OR “Cytarabine” [Mesh])) AND (((((Primary central nervous system lymphoma) OR PCNSL) OR Primary CNS lymphoma) OR Primary central lymphoma))). In addition, we searched the reference lists of the identified articles and previous meta-analysis to identify other potential studies.

### Inclusion and Exclusion Criteria

Articles were considered eligible if they met the following criteria: (1) randomized clinical trials (RCTs); (2) the patients were newly diagnosed with PCNSL and confirmed by histopathology; (3) sufficient survival outcome data must be reported such as CR, ORR, PFS (FFS), and OS; (4) grade 3–4 toxic effects of treatment must be reported; (5) the aim of the study was to compare between treatment of PCNSL patients with or without cytarabine; and (6) cytarabine was used systemically and not intrathecally.

Studies were excluded based on the following criteria: (1) written in a language other than English; (2) one-arm clinical trials; (3) reviews, letters, reports, conference abstracts or papers, mail articles, and editorials; (4) sample cases from a database; and (5) not related to the topic.

### Data Extraction

Two investigators (ZXH and YSB) independently extracted the data from the included studies using a standardized form. Data extraction was performed according to the method of Song et al. (8) and included the following items: (1) last name of the first author, publication year, and study design; (2) study population location, sample number, and intervention; (3) dose and cycle of cytarabine; and (4) survival outcomes including CR, ORR (including complete remission rate and partial remission rate), PFS, and OS. The data were directly collected from the article if the hazard ratios (HRs), 95% confidence intervals (95% CI), and *P*-value were reported; otherwise, we contacted the corresponding authors to obtain these data or extracted the data from Kaplan–Meier curves using Engauge Digitizer version 13.0 (9).

### Statistical Analysis

Statistical analysis was independently performed by two investigators (ZXH and YSB) using Stata 12.0 (StataCorp, College Station, TX, USA) and RevMan 5.3. Disagreements were resolved by a third investigator (Chen). Heterogeneity among the studies was assessed using the Chi-square test (*Q*-statistic) and *I*^2^ statistic; if *P* ≥ 0.10 and/or *I*^2^ < 50%, a fixed-effect (Mantel–Haenszel method) model was used as the heterogeneity was recognized as being low; otherwise, a random effects (Mantel–Haenszel method) model was used because of significant heterogeneity. The effect size (ES) for each meta-analysis was calculated as follows: (1) CR rate, overall ORR, and grade 3–4 toxic effects rate: odds ratios (ORs) with corresponding 95% confidence interval (CI) and (2) PFS and OS: HRs and a 95% CI. For the CR rate and the ORR, PFS, and OS, if OR > 1.0, HR < 1.0, and *P* < 0.05, the results favored the combined cytarabine therapy group and were considered statistically significant. For the grade 3–4 toxic effects rate, if OR < 1.0 and *P* < 0.05, the results favored the therapy with cytarabine group and was considered statistically significant. In addition, we conducted quality assessment (by Cochrane Collaboration risk of bias tool) and sensitivity analysis (by omitting any single study).

## Results

### Characteristics of the Studies

**Figure 1:**
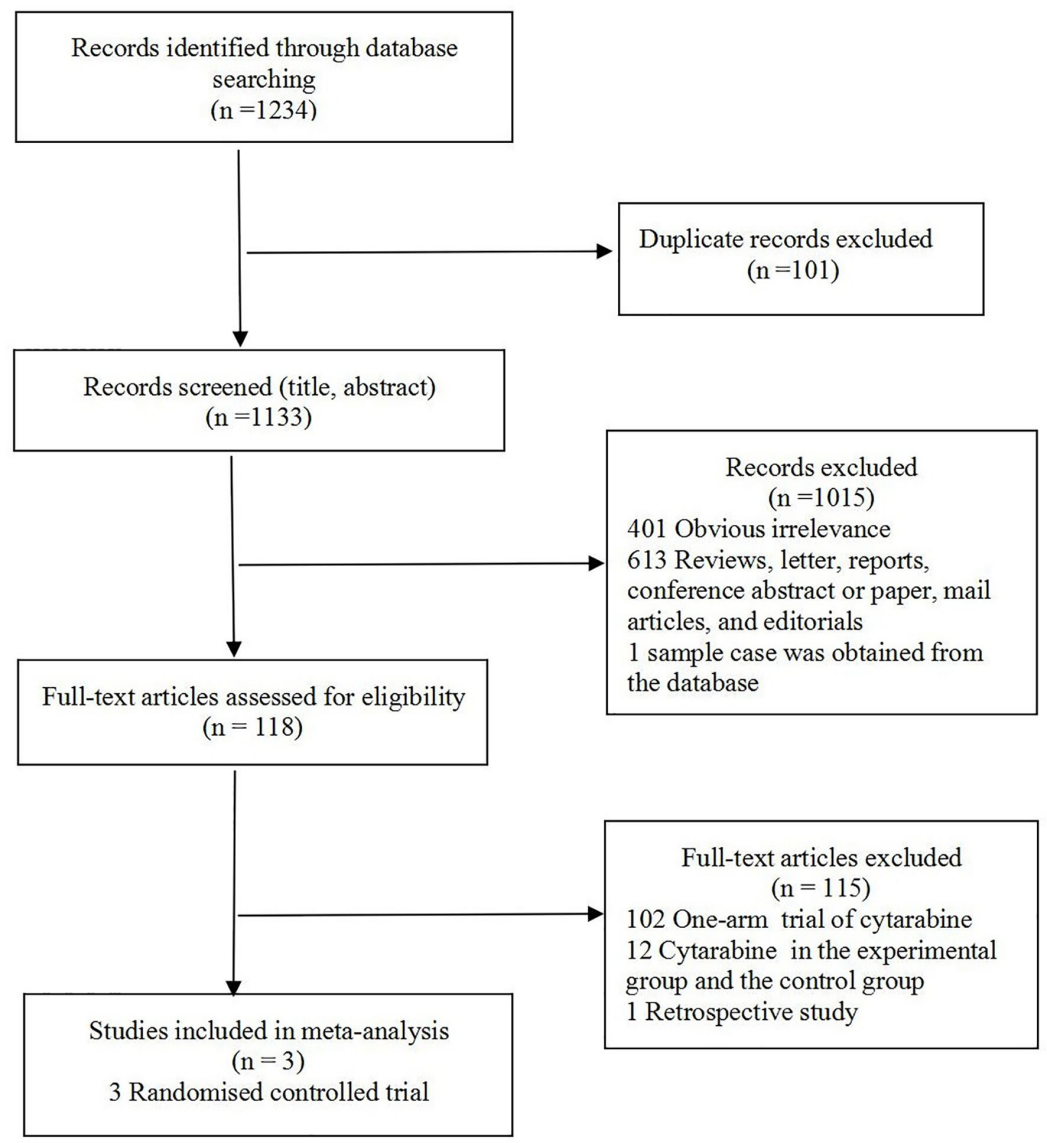
Flow diagram of the study selection process.

The study selection flowchart is shown in Figure 1. At the end of selection, three RCTs (4, 6, 10) involving 223 participants were included in the meta-analysis. The classification and features of the included studies are shown in Table 1, including study design, publication year, recruitment period, location, age, sample size, intervention, cytarabine dosage, and outcome indicators. The three studies were conducted in different countries: one in six countries (Argentina, Greece, Italy, Peru, Portugal, and Switzerland), one in France, and one in China. Upon further review, three articles contained data of CR and ORR. Omuro et al. (10) showed that the response (CR, ORR) of 87/95 randomized patients could be evaluated; five patients died from toxicity before the first magnetic resonance imaging (MRI), one patient could no longer receive contrast, and two withdrew consent.

**Table 1:** Characteristics of enrolled studies.

**References**	**Study design**	**Year**	**Recruitment period**	**Location**	**Age**	**Sample size Ara:non-Ara**	**Intervention Ara:non-Ara**	**Dosage of Ara**	**Cycles of Ara**	**Outcome indicators**
(6)	RCT	2018	2012–2015	China	14–69	25:24	MTX+Ara-C+WBRT:FTD+WBRT	1 mg/m^2^	4	CR, ORR, PFS, OS
(10)	RCT	2015	2007–2010	France	>60	47:48	MTX+Ara-C+PV:MTX+TMZ	3 mg/m^2^	3	CR, ORR, PFS, OS
(4)	RCT	2009	2004–2007	Argentina, Greece, Italy, Peru, Portugal, Switzerland	18–75	39:40	MTX+Ara-C+WBRT:MTX+WBRT	2 mg/m^2^	4	CR, ORR, FFS, OS

*Ara, treatment with cytarabine; non-Ara, treatment without cytarabine; RCT, randomized clinical trial; MTX, methotrexate; Ara-C, cytarabine; WBRT, whole-brain radiotherapy; FTD, fotemustine, teniposide, and dexamethasone; PV, procarbazine and vincristine; CR, complete remission; ORR, overall response rate; OS, overall survival; PFS, progression-free survival; FFS, failure-free survival*.

In total, 223 patients were included in the studies we examined, of which 111 patients received therapy with cytarabine and 112 patients received non-cytarabine therapy. The three studies compared cytarabine with non-cytarabine treatment in patients with newly diagnosed PCNSL. In terms of therapeutic scheduled chemotherapy, the MTX-based regimen was the most common therapeutic regimen. Combination chemotherapy without HD-MTX and whole-brain radiotherapy (WBRT) were also reported.

### Meta-Analysis of CR, ORR, Grade 3–4 Toxic Effect Rate, PFS, and OS Rate for Treatment With or Without Cytarabine

**Figure 2:**
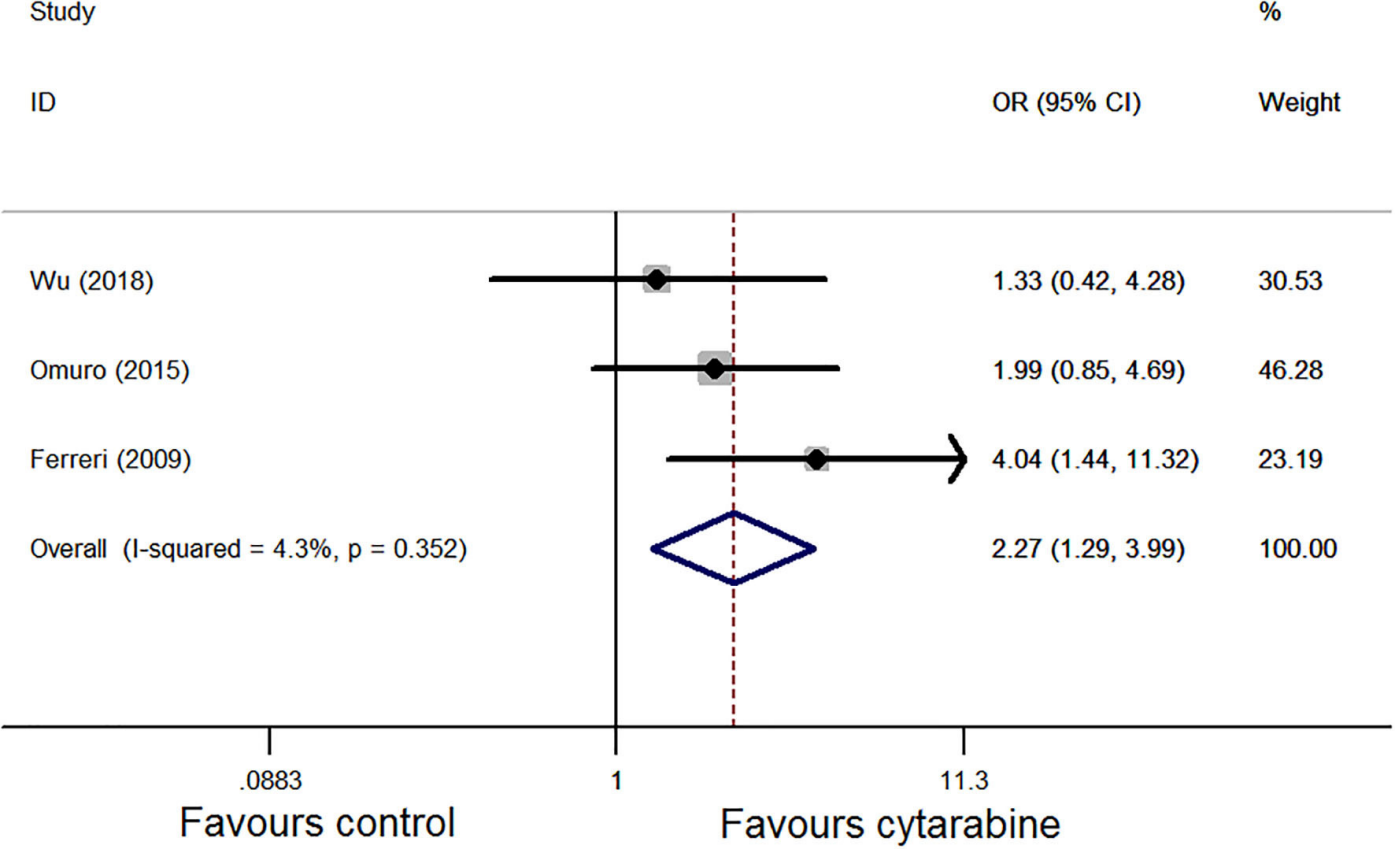
Forest plot of the complete remission for treatment with the cytarabine group vs. cytarabine-free group (fixed effect model). OR is the effect size.

We chose the Mantel–Haenszel fixed model for CR analysis because of the observed heterogeneity among the included studies (*P* = 0.35, *I*^2^ = 4.3%; Figure 2). The cytarabine group was associated with a better outcome (OR: 2.27, 95% CI: 1.29-3.99, *P* < 0.01).

**Figure 3:**
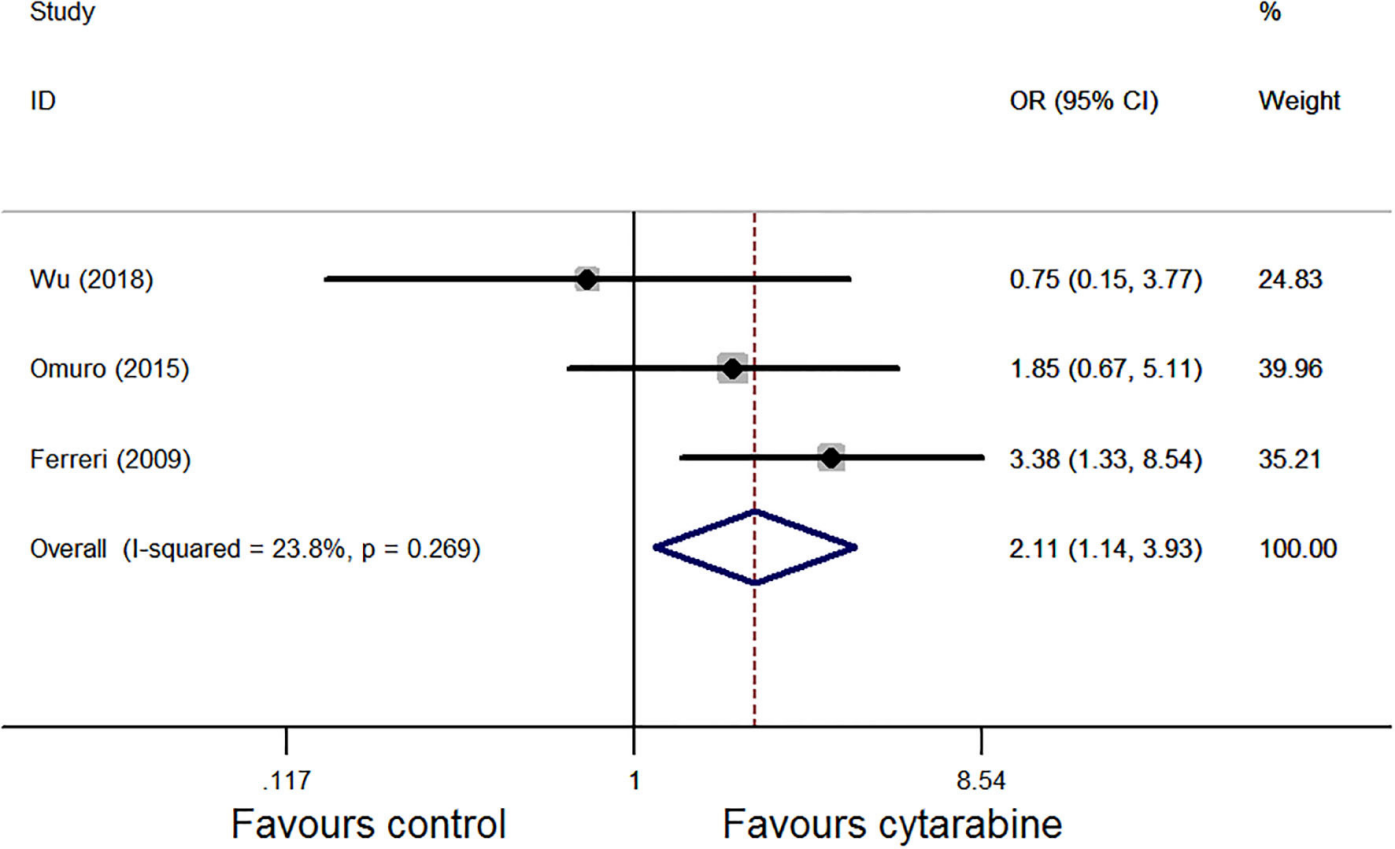
Forest plot of the overall response rate for treatment with the cytarabine group vs. cytarabine-free group (randomized effect model). OR is the effect size.

We also used the Mantel–Haenszel fixed model for ORR analysis as heterogeneity was observed (*P* = 0.27, *I*^2^ = 23.8%; Figure 3); the results favored the cytarabine group (OR: 2.11, 95% CI: 1.14–3.93, *P* = 0.02).

**Figure 4:**
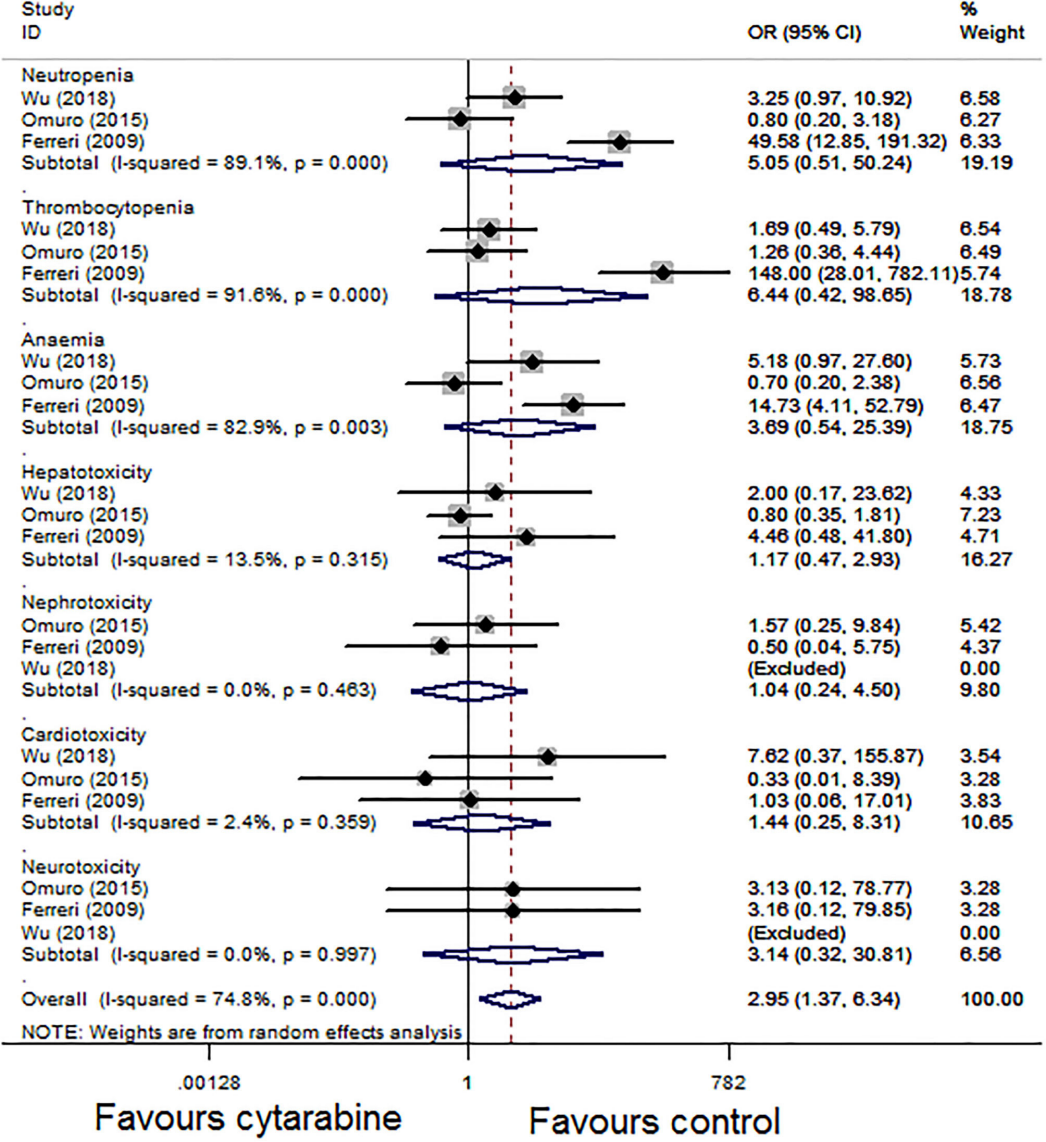
Forest plot of grade 3–4 toxicities for treatment with the cytarabine group vs. the cytarabine-free group (fixed effect model). OR is the effect size.

For the grade 3–4 toxic effect rate, we performed a subgroup analysis of the three studies based on the type of side effect. The pooled result of the three studies showed that the grade 3–4 side effect rate of the cytarabine group was higher (overall OR: 2.95, 95% CI: 1.37–6.34, *P* < 0.01), with high heterogeneity (*P* < 0.01, *I*^2^ = 74.8%; Figure 4).

**Figure 5:**
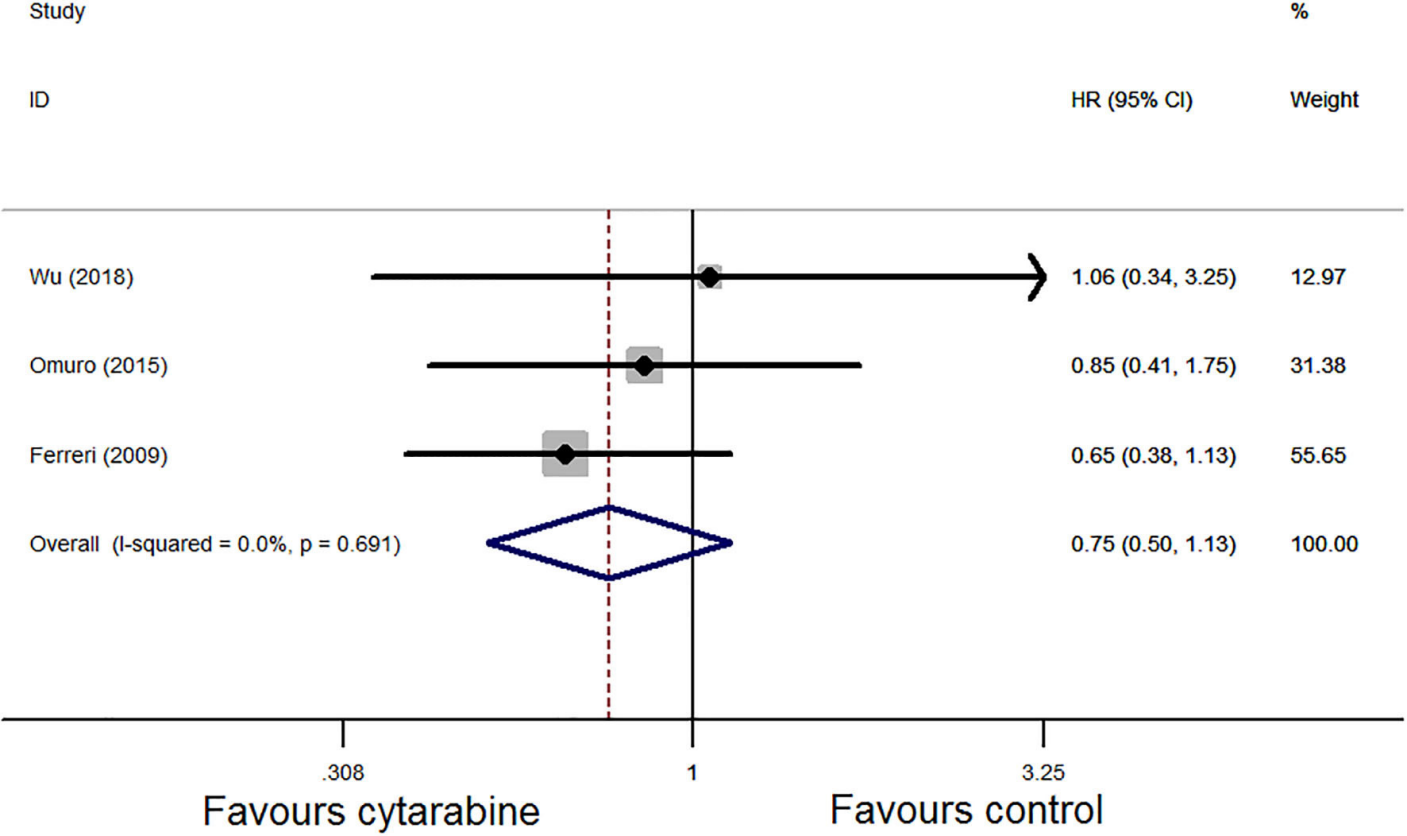
Forest plot of overall survival for treatment with the cytarabine group vs. the cytarabine-free group (fixed effect model). HR is the effect size.

For the OS, three studies assessed the HR of the cytarabine group vs. the cytarabine-free group. Pooling the data of these studies showed no significant difference in the OS (HR: 0.75, 95% CI: 0.50–1.13, *P* = 0.17), with no significant between-study heterogeneity (*P* = 0.69, *I*^2^ = 0.0%; Figure 5).

**Figure 6:**
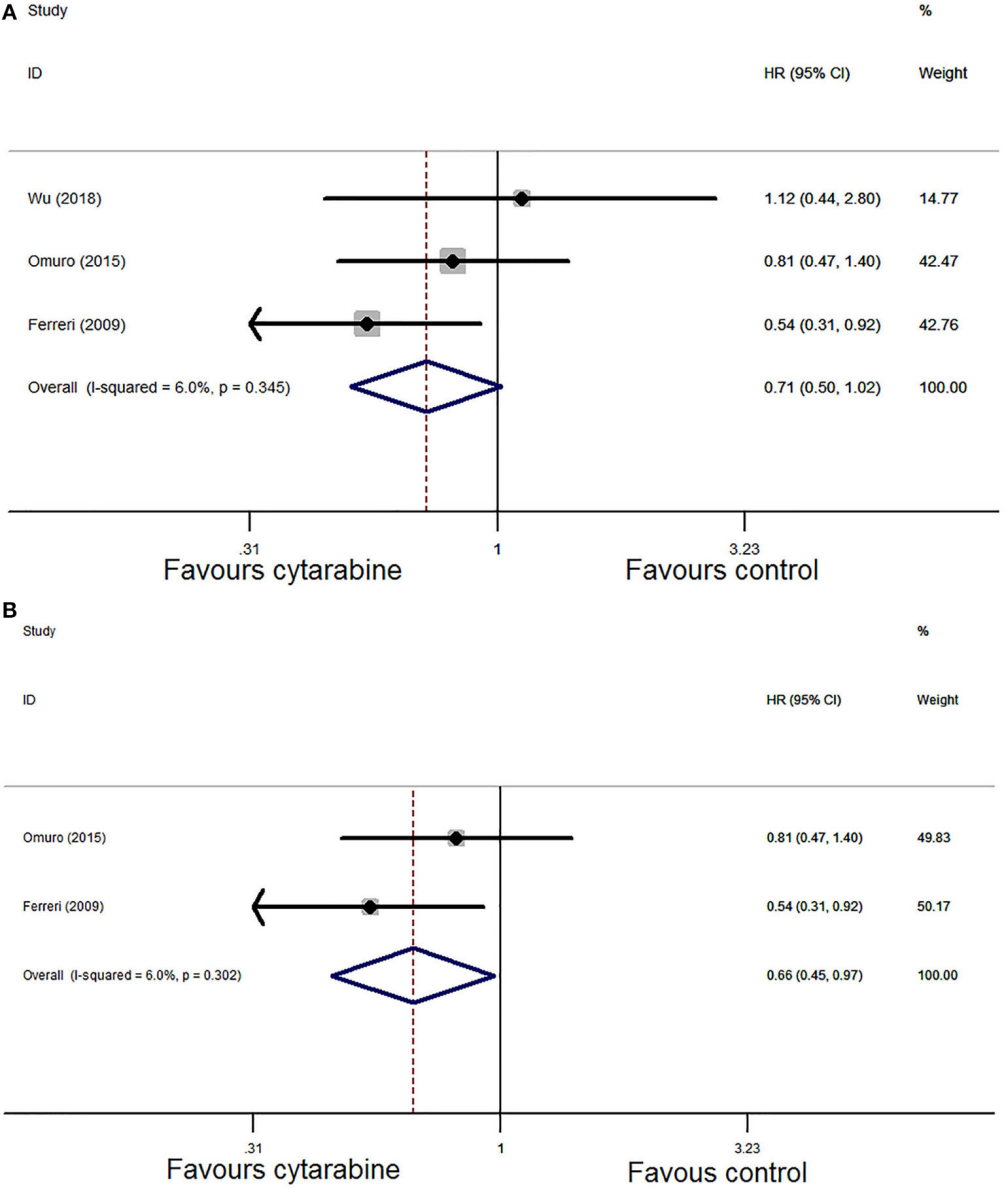
**(A)** Forest plot of progression-free survival for treatment with the cytarabine group vs. the cytarabine-free group (fixed effect model). HR is the effect size. **(B)** Forest plot of progression-free survival after deleting one study (fixed effect model). HR is the effect size.

Pooled analysis of PFS in the three trials demonstrated that the HR was 0.71 (95% CI: 0.50–1.02, *P* = 0.06). We chose the Mantel–Haenszel fixed model based on the observed heterogeneity among the chosen studies *(P* = 0.35, *I*^2^ = 6.0%; Figure 6A). In the sensitivity analysis, the combined result of PFS was unstable. Then, we eliminated one study in low-quality and recombined HR which was 0.66 (95% CI: 0.45-0.97, *P* = 0.04) with no significant between-study heterogeneity (*P* = 0.30, *I*^2^ = 6.0%; Figure 6B). The results favored the cytarabine group.

### Quality Assessment

The quality of the included studies was independently assessed by two investigators (Zheng and Yang). We used the Cochrane Collaboration risk of bias tool to evaluate the included RCTs according to the Cochrane Handbook recommendations. The items included random sequence generation (selection bias), allocation concealment (selection bias), blinding of participants and personnel (performance bias), blinding of outcome assessment (detection bias), incomplete outcome data (attrition bias), selective reporting (reporting bias), and other biases. The risk of bias graph and risk of bias summary showed that two of the three RCTs (4, 10) included were high-quality studies and one of the studies (6) was of low quality (Supplementary Figures S1A,B).

### Sensitivity Analysis and Publication Bias

We conducted sensitivity analysis by eliminating individual studies one at a time to check the influence of the removed data set to the overall effect size. The results of the sensitivity analysis indicated that the pooled result was stable in CR, OR, toxic effect, and OS analysis except PFS (Supplementary Figures S2–S5). We removed each of the studies, and the overall results of PFS had significant statistical difference after removing the study by Wu et al. (6), which showed that the combined result was unstable in PFS analysis (Supplementary Figure S6). So this study was deleted in PFS analysis and HR was re-pooled. As our analysis included fewer than 10 studies, we did not conduct a publication bias analysis.

## Discussion

Untreated PCNSL has a dismal prognosis with a median survival of ~3 months. Although the outcome of patients with newly diagnosed PCNSL has improved owing to the progress of treatment, prospective clinical trials are rare and the optimal treatment for PCNSL is uncertain. HD-MTX is a cornerstone of PCNSL treatment (11). HD-MTX-based chemotherapy combined with radiotherapy is a traditional treatment strategy for PCNSL. Although some studies have reported that the combination of MTX-based regimens with WBRT has promising effects, it leads to an increased incidence of advanced neurotoxicity (12, 13). Hence, WBRT is no longer a routinely recommended treatment for patients with newly diagnosed PCNSL. Recently, the approach omitting WBRT is high-dose chemotherapy followed by autologous stem cell transplantation (HDT/ASCT), which has shown encouraging results, especially for young patients with PCNSL (age <65 years) (14, 15). However, transplantation-related toxicities have also caused extensive concern among neurooncologists.

The optimal treatment of PCNSL has yet to be defined. Most studies investigating systematic therapy of PCNSL advocate the use of HD-MTX in combination with other chemotherapeutic or target drugs, such as cytarabine (5), temozolomide (16, 17), and rituximab (18, 19), to improve patient survival. These drugs have been selected based on their ability to penetrate the blood–brain barrier and their efficacy against systemic lymphomas. Among them, cytarabine is a relatively commonly used treatment option.

Cytarabine is a medium-permeable drug based on the extent to which the drug passes through the blood–brain barrier (20). An international retrospective study involving 378 PCNSL patients (11) revealed that survival improvement resulted from the addition of high-dose cytarabine to HD-MTX. In 2009, the International Extranodal Lymphoma Study Group (IELSG) reported the first randomized study involving immunocompetent patients with PCNSL (IELSG 20) (4). A significant increase in complete remission rate and failure-free survival (FFS) were observed when high-dose cytarabine was combined with HD-MTX. Therefore, high-dose cytarabine is widely used in the treatment of PCNSL.

The retrospective study conducted by Wang et al. (7), however, suggested that both PFS and OS were not higher in the with-cytarabine group compared with a therapeutic regimen without cytarabine. Furthermore, an RCT conducted by Wu et al. (6) demonstrated that the non-cytarabine group appeared to be safer and more effective for PCNSL patients compared with the cytarabine group. These results were inconsistent with the previous studies (4, 11). It is possible that these contradictory results are due to different backbone therapy options. Moreover, it shows the urgent necessity of assessing the effect of cytarabine on newly diagnosed PCNSL patients.

This meta-analysis was conducted to evaluate the effect and safety of cytarabine on newly diagnosed PCNSL patients. As the rarity of these tumors hindered the performance of randomized trials, few RCTs were included in our analysis, and a comparison between therapeutic approaches for PCNSL with or without cytarabine was made. To the best of our knowledge, this study is the first meta-analysis to evaluate the role of cytarabine in PCNSL treatment.

Our results showed that combined treatment with cytarabine significantly improves the CR rate (*P* < 0.05) and ORR (*P* < 0.05). The side effect rate of the cytarabine group is higher (*P* < 0.05). We conducted a subgroup analysis based on the types of side effect; grade 3–4 toxicity was more common in the cytarabine group, especially hematological toxicity (neutropenia, thrombocytopenia, and anemia). In the study conducted by Ferrier et al. (4), neutropenia, thrombocytopenia, and anemia (the OR were 49.58, 148.00, and 14.73, respectively, Figure 4) were recorded in most patients of the combined cytarabine group. Neurotoxicity, as a common side effect after many treatments of brain tumors, has aroused extensive concern of neurooncologists. Nonetheless, the incidence of neurotoxicity did not increase when cytarabine was added in our analysis. Although the pooled effect estimates did not show evidence for improvement of OS (*P* > 0.05), the addition of high-dose cytarabine to treatment did provide improved PFS (*P* < 0.05). Those above results illustrated the effects and feasibility of the drug in the induction treatment of PCNSL.

According to the quality assessment results, one of the studies (6) was of low quality. The reason may be related to the small sample size and the large age span of the recruited patients. Then, we performed the sensitivity analysis by eliminating individual studies one at a time to check the stability of the overall results. We found that the results of CR, OR, toxic effects, and OS were stable. However, the result of PFS was unstable. One of the studies (6) was found to affect the final pooled results of PFS. We deleted this study and re-pooled the results (data of 2 RCT). The new result indicated that the PFS was improved in the cytarabine arm. There was no significant heterogeneity among the individual studies in terms CR, ORR, PFS, and OS, suggesting that the backgrounds of the eligible studies were of good homogeneity.

The guidelines of the National Comprehensive Cancer Network (NCCN) have recommended rituximab as the first-line treatment regimen for diffuse large B-cell lymphoma. Although >95% of PCNSLs are diffuse large B-cell lymphomas (21), the combination of rituximab with chemotherapy for PCNSL remains controversial (22). Several retrospective and single-arm prospective studies have indicated a favorable effect for rituximab on PCNSL outcome. Furthermore, a randomized study (IELSG 32) (19) of 227 patients also suggested a survival improvement resulting from a combined rituximab therapeutic regimen in patients with newly diagnosed PCNSL. However, according to a recent large, randomized intergroup phase 3 study (23) involving 200 patients, no clear benefit was observed for the addition of rituximab to chemotherapy in PCNSL. None of the three studies included in our analysis contained rituximab. The clinical significance of adding cytarabine on the basis of rituximab remains to be further studied.

Great breakthroughs have been made in the treatment of PCNSL in recent years, such as ibrutinib (24), lenalidomide (25), and CAR-T cells (26). However, most of them are still based on small samples of phase I/II trials. Further large RCTs are required to verify the effects and safety of those drugs. Many ongoing randomized trials evaluate different approaches using HD-MTX-based chemotherapy with treatment like ibrutinib, pemetrexed, lenalidomide, and CAR-T cells, which may bring new light to the treatment of PCNSL.

### Limitations

A limitation of this analysis is that few RCTs were included. It is difficult to conduct a large-sample RCT study because of the rarity of PCNSL. To some extent, the limited sample number may influence the strength of our analysis. Second, some data extracted from the Kaplan–Meier curve may have been biased. Third, there were significant differences in the backbone therapy used among the different institutions including chemotherapy or radiotherapy regimens. For example, the treatment of one study (10) did not contain WBRT; the control group therapeutic regimen of one study (6) did not combine MTX. Fourth, we did not examine publication bias because the number of included studies was insufficient.

## Conclusions

This meta-analysis verified that adding cytarabine to the therapeutic regimen is of great benefit to newly diagnosed PCNSL patients in terms of CR, ORR, and PFS. In addition, it should be noted that the grade 3–4 toxic effects, especially hematological toxicity, are higher in the cytarabine group. Few fatal toxic effects occurred in the study, confirming the excellent safety of adding cytarabine to methotrexate-based chemotherapy. Our study provides a theoretical basis about adding cytarabine in the induction therapy of PCNSL. Large-sample and high-quality RCTs should be conducted to verify our results and confirm the effects of cytarabine on newly diagnosed PCNSL. Further studies are required to determine the optimal dosage and schedule of cytarabine in the treatment of PCNSL.

## Data Availability Statement

All datasets presented in this study are included in the article/Supplementary Material.

## Author Contributions

FC and WL conceived the topic of the study and critically revised it for important intellectual content. XZ searched the literature, analyzed the data, created the figures, and drafted the manuscript. SY searched the literature, analyzed and interpreted the data, and assisted in writing the manuscript. SW undertook the task of drafting and proofreading the manuscript. All authors contributed to the article and approved the submitted version.

## Conflict of Interest

The authors declare that the research was conducted in the absence of any commercial or financial relationships that could be construed as a potential conflict of interest.
