# Supplementary material for: The Efficacy and Safety of Cytarabine on Newly Diagnosed Primary Central Nervous System Lymphoma: A Systematic Review and Meta-Analysis
Source: Front Oncol. 2020 Jul 31;10:1213. doi: 10.3389/fonc.2020.01213 (PMC7438862; doi:10.3389/fonc.2020.01213)
Supplement: Supplementary file 1 [file Data_Sheet_1.docx]

**Supplementary Figure**


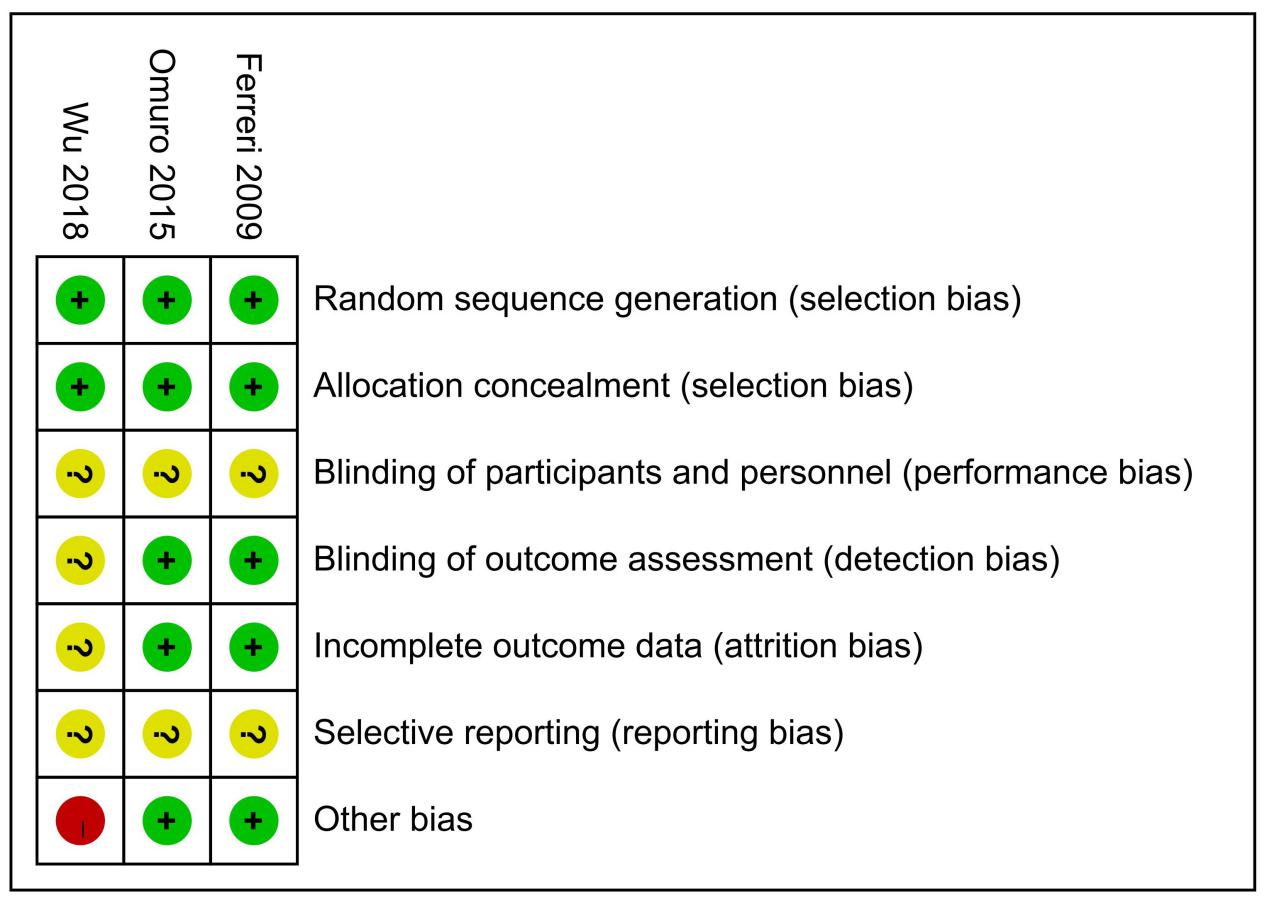


Supplementary Figure S1 (A) Risk of bias summary.


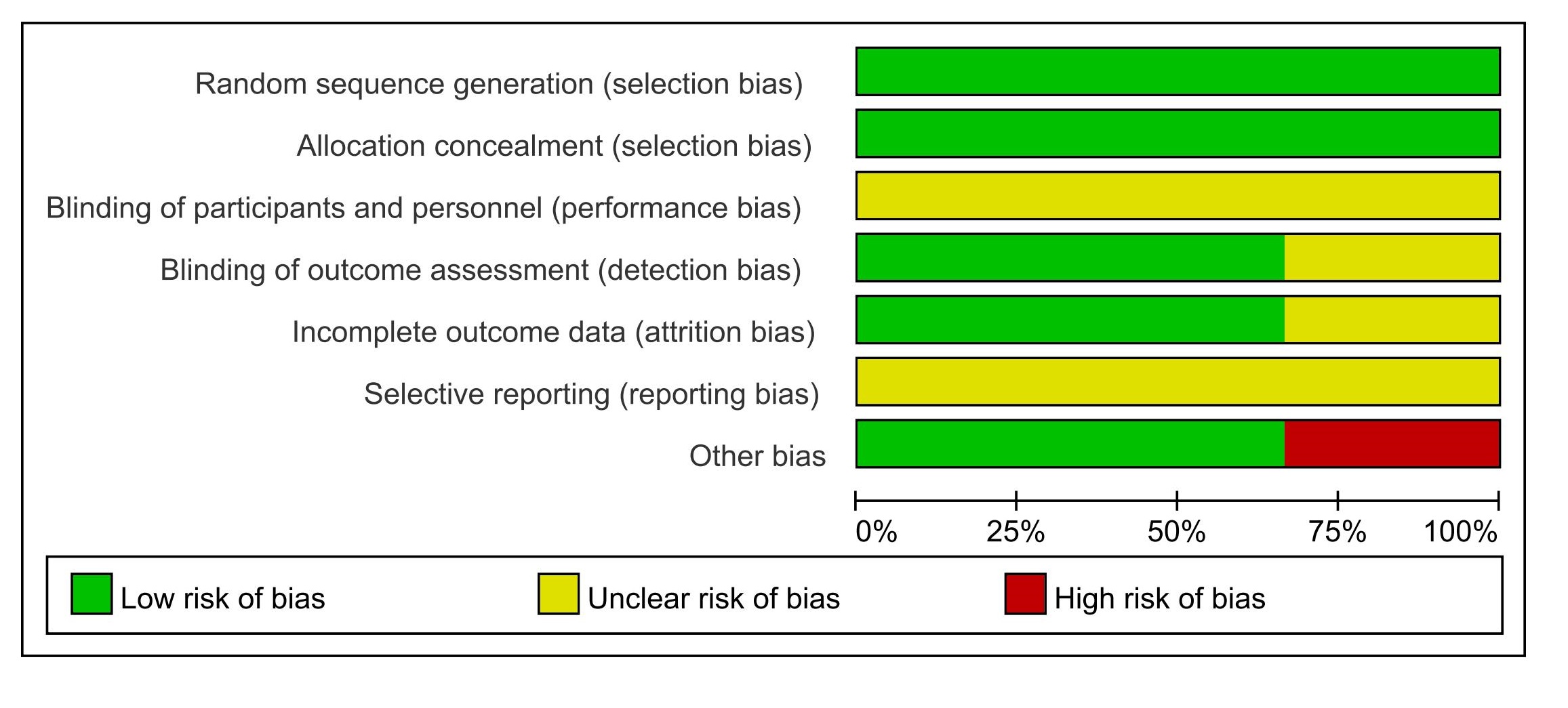


Supplementary Figure S1(B) Risk of bias graph.


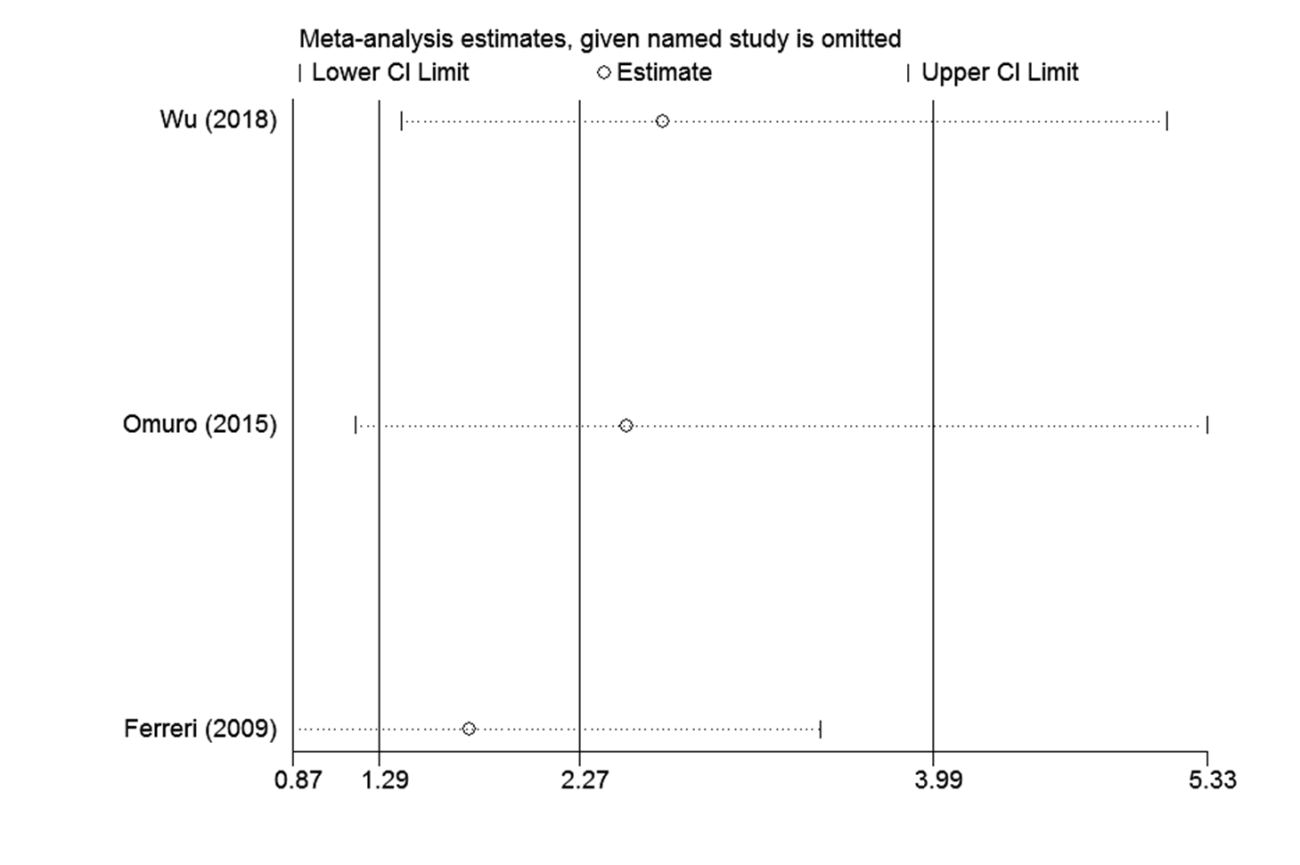


Supplementary Figure S2 The sensitivity analysis of the complete remission.


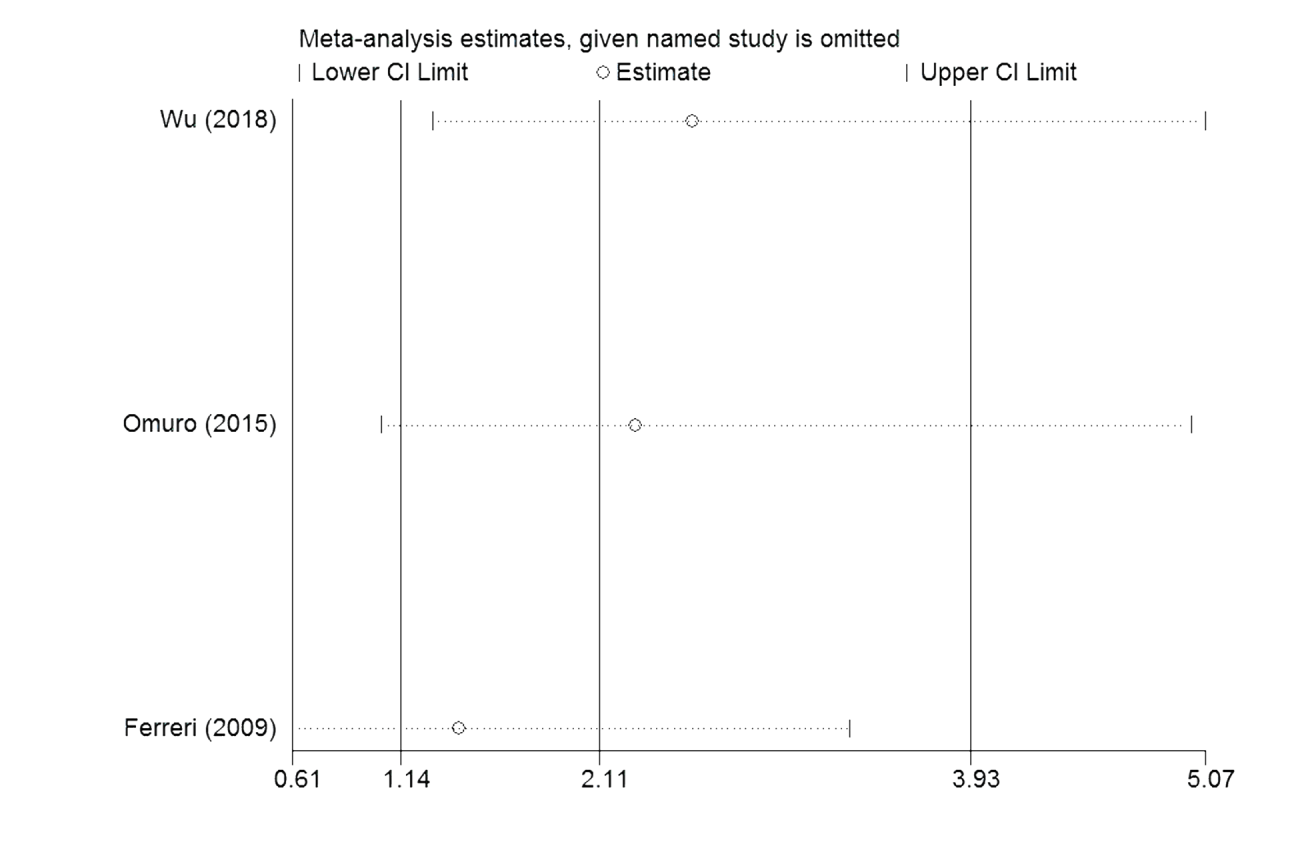


Supplementary Figure S3 The sensitivity analysis of the overall response rate.


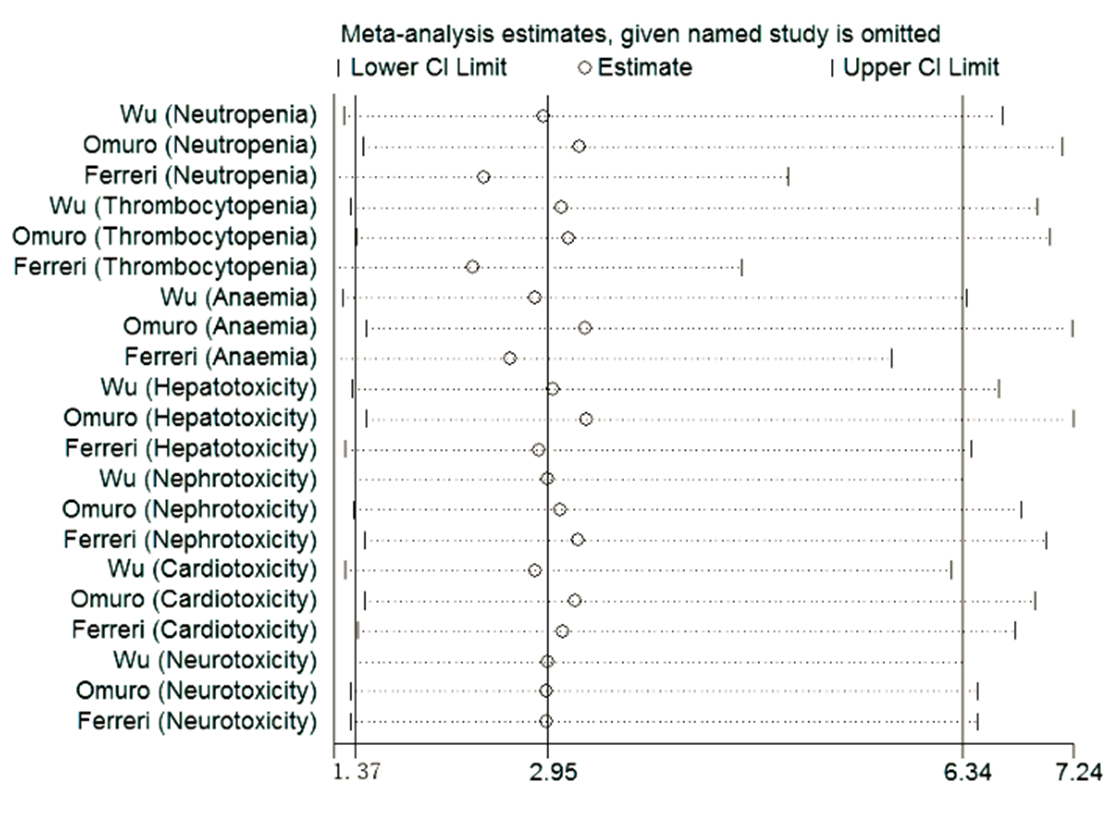


Supplementary Figure S4 The sensitivity analysis of the grade 3-4 toxic effects rate.


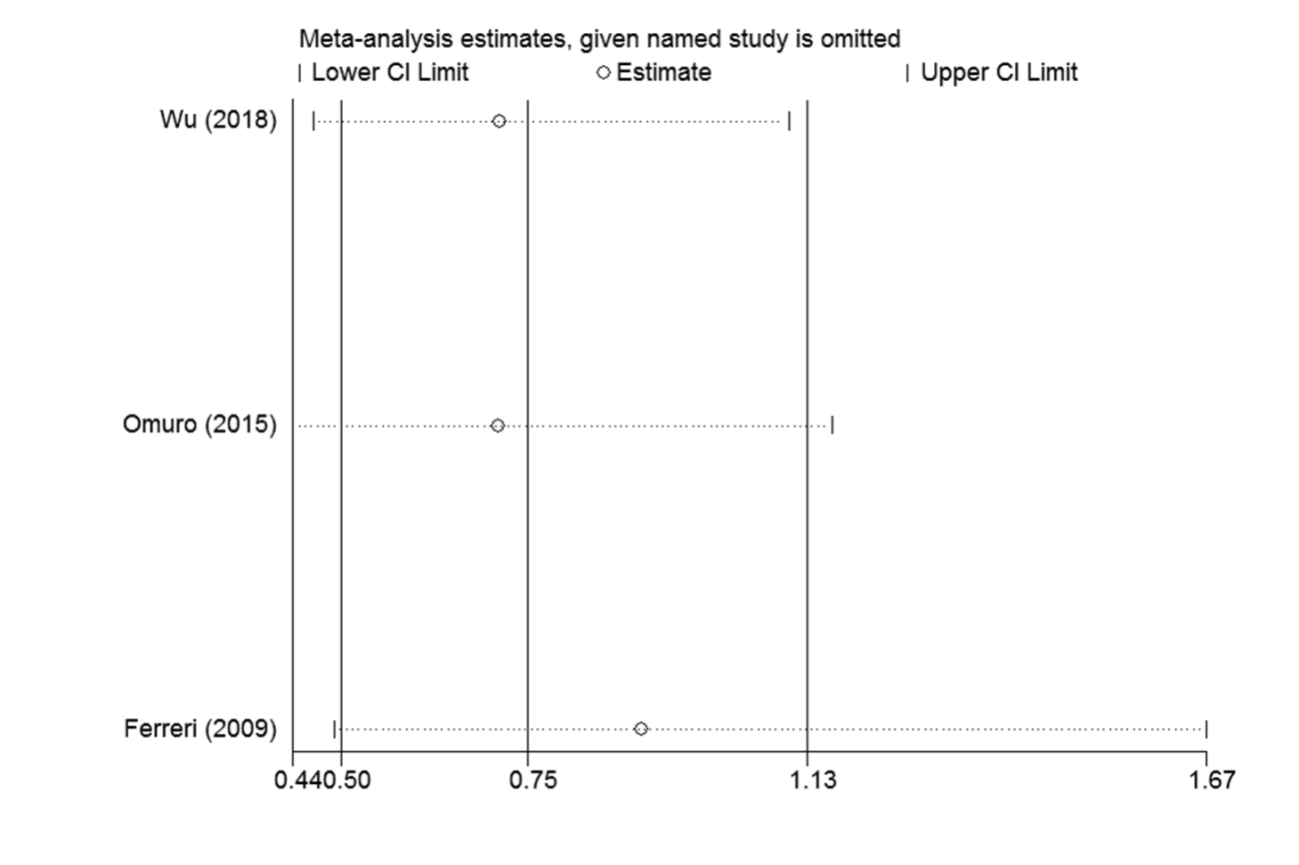


Supplementary Figure S5 The sensitivity analysis of overall survival.


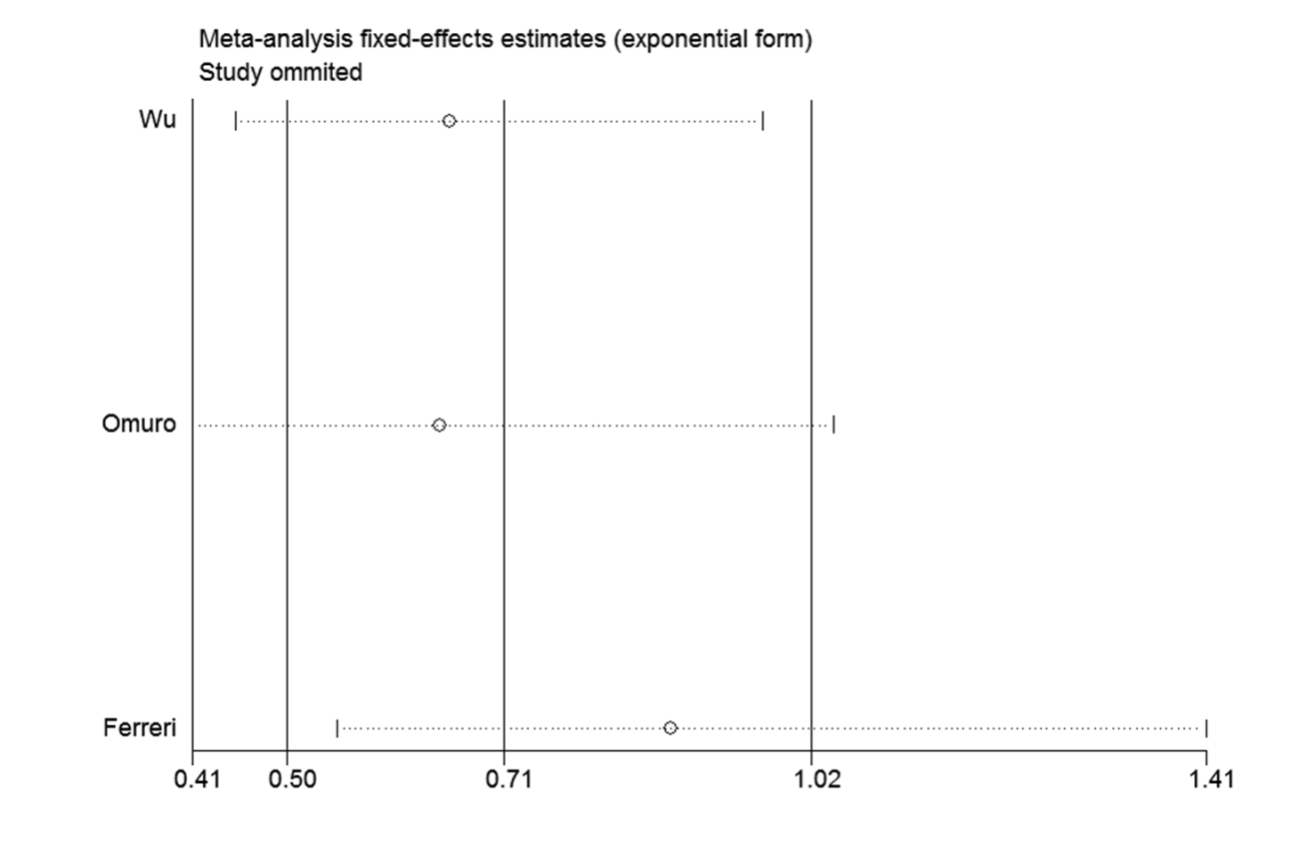


Supplementary Figure S6 The sensitivity analysis of progression-free survival.
